# Report from the second MIB (Melanoma Independent Board) Conference, 27–28 October 2014

**DOI:** 10.3332/ecancer.2015.511

**Published:** 2015-02-19

**Authors:** Alessandro Testori, Carlo Riccardo Rossi, Paolo Ascierto, Francesco de Lorenzo, Nicola Mozzillo

**Affiliations:** 1 European Institute of Oncology, Via Ripamonti 435, Milan 20141, Italy; 2 Istituto Oncologico Veneto, Padova, Italy; 3 Istituto Nazionale dei Tumori di Napoli, Italy; 4 Federazione Italiana delle Associazioni di Volontariato in Oncologia, Italy

**Keywords:** innovation, sustainability, melanoma multidisciplinarity, patients associations

## Abstract

The second Melanoma Independent Board (MIB) meeting was held in Rome, Italy from 27–28 October 2014. The themes of this meeting were once again innovation and sustainability and it brought together health providers, researchers, government representatives, journalists, patients, and pharmaceutical representatives.

## Introduction

Innovation and sustainability were the themes of the second annual MIB meeting, which was held in Rome from 27–28 October, 2014. Innovation was spoken of in terms of not only new drugs and treatments, but also regarding the organisation of treatment facilities, particularly in Italy.

In addition, the meeting addressed the sustainability in providing the best available treatment to all patients, even as new drugs become available and the cost of treatment increases. Intertwined with both of these main themes were the patients themselves. This meeting paid particular attention on how decisions and cost analysis affected the patient and there were also a number of talks that considered the perspective of a patient.

### Session 1

The first session of the conference opened with some welcoming remarks on behalf of regional and institutional leaders in Italy. As the host of the event, Dr Alessandro Testori, of the Istituto Europeo de Oncologia, Milano (IEO) gave the opening talk, which focused on the impetus for these meetings as well as of his upcoming project.

As it was last year, this year’s meeting was dedicated to the memory of Pino Casenelli, one of the pioneers in melanoma research [1]. Dr Testori noted that melanoma is one of the more innovative diseases that we have to deal with in oncology and through Dr Caenelli’s work we too have started a process of innovation. However, it is the communication and dissemination of this research that is important [2]. Dr Testori stated that our goal must be to reach a mortality of zero in melanoma, and he also highlighted some of the challenges melanoma carers faced, including the need for better technology to improve diagnosis, the importance of further education for the population as a whole and also that of primary care doctors.

Dr Testori then turned his attention to an epidermatology project he is working on that may help provide an earlier diagnosis of melanoma. The project, sponsored by Vodafone, can be considered a scientific validation of teledermatology. It tests the ability to diagnose suspicious lesions with simple photographs taken from a mobile phone. Melanoma doctors from seven institutes around Europe are participating in the study where 1000 patients who will be operated on for a skin lesion (either benign or tumoural) take images of four moles on their skin, including the one to be operated on. Two doctors, chosen randomly from within the seven institutes, then assess the moles from these images. Dr Testori further outlined details of the image diagnosis and the primary and secondary endpoints of this study. It is hoped that this study will get final approval soon.

Aldo Morrone was next to speak with a greeting from the health ministry in Italy. His talk focused on some melanoma work he had done in Africa, particularly in Ethiopia, over the last 30 years. Dr Morrone pointed out that skin cancer has traditionally been assumed to be infrequent in dark-skinned individuals, but there has been little data available concerning its actual prevalence and incidence; most of the data available on melanoma in Black individuals comes from African Americans. Unlike people of European descent, for whom basal cell carcinoma is most common, squamous cell carcinoma (SCC) is the most frequent type of non-melanoma skin cancer among dark-skinned people, making up to 60% of all cases of skin neoplasm [3].

In 1985, Dr Morrone helped open an institute dedicated to melanoma care in Ethiopia. During the past 30 years they have learned that there is a lot of melanoma and non-melanoma skin cancer in Ethiopia. From 2005 until 2013, more than 54,000 patients were observed in their centre. Of this, a total of 1365 patients presented with 1392 skin tumours (2.4%), which could be classified into 15 categories. Of these, one quarter were malignant tumours (347) of the skin and the mucous membranes. He concluded his talk by highlighting the need for education within the community and among health professionals to increase the possibility for a favourable outcome.

Following this talk, further welcome messages were presented by Maria T. Balldini, on behalf of the Lombardy region, Antonello Aurigemma, on behalf of the Lazio region and Pierpaolo Cavallo on behalf of Federlab Italia.

Gordon McVie, from the IEO and *e*cancer, then gave a talk on his work through *e*cancer. He noted that communicating progress in research and care must now be done online, through the internet. He pointed out that most patients nowadays go online for information and that most young doctors read scientific journals online, not paper copies. McVie went on to highlight some of the goals and achievements of *e*cancer.

Sara Gandini, also from the IEO in Milan, then provided an introduction on the epidemiology of the current impact of melanoma worldwide. She noted that within the EU there are 2.66 million new cancers diagnosed every year with 1.28 million cancer-related deaths. Of these, there are 100,000 new cases of melanoma and 22,000 deaths in Europe [4]. The UN estimates a global increase in the incidence of melanoma over the next few years; by 2020 it is predicted that there will be 280,000 new cases of melanoma worldwide and 68,000 deaths per year [4].

Dr Gandini then highlighted some incidence trends and prevention strategies. Sunbed use is now considered one of the greatest preventable risks of melanoma, with one study reporting a 1.8% increase in the risk of melanoma after each sunbed session [5] UV protection, especially for children, was noted as one of the most important methods of decreasing risk of melanoma. Melanoma is also considered a disease of the young and middle aged, with cutaneous melanoma commonly being diagnosed in patients between 40 and 60 years of age and melanoma being the most common cancer in people aged 25–29 [4]. Socioeconomic trends for both incidence and survival were also highlighted as were country-based cases, with Australia, New Zealand, USA, Canada, Norway showing signs of a plateau in diagnosis while other countries, including Denmark, England, Spain and Italy, continue to have increased diagnosis every year. Gandini then reported his observation on the status of melanoma patients based on their socioeconomic status. While individuals of higher socioeconomic status are more likely to be diagnosed with melanoma, they have a higher survival rate than those individuals of lower socioeconomic status.

Next, Dr Enrico Proietti presented a talk on the role of Istituto Superiore di Sanita (ISS) in supporting Italian clinical research projects.The Institute conducts scientific research in a wide variety of fields, from cutting-edge molecular and genetic research to population-based studies on risk factors for disease and disability. Research priorities are based on those set forth in the National Health Plan. The Institute is also involved in several major clinical trials, which are frequently conducted in cooperation with the Scientific Institutes for Research and Care (IRCCS) network and hospitals.

Maurizio de Cicco, Vice President of Farmindustria, presented some of the challenges being faced by pharmaceutical companies. He started off by noting the positive impact drugs and new treatment techniques have had on patient survival. Overall, in the last 25 years life expectancy has increased by at least 4–5 years. However, this increase in years comes at a cost: every second, the public debt in Italy increases by 2000 euros. From there he looked at the regulations regarding drug cost and approval. He noted that pharmaceutical spending is the most regulated in a sector. In addition new drugs take nearly two years before they are approved for use in Italy and there is then an additional year before Italy offers reimbursement. These issues limit access to potentially life-saving drugs.

De Cicco then briefly discussed the new registry, which is being implemented in Italy. While the new registry has the potential to improve patient diagnosis and care, there are currently a number of issues with the new registry system. He commented on the importance of getting the registry working.

Francesco de Lorenzo, President of the European Patient Coalition (ECPC), highlighted the challenges patients face as governments and healthcare providers deal with the issue of sustainability and the threat of cuts. He noted that we may find ourselves in a situation where a cancer patient who could once be cured can now only receive palliative care. De Lorenzo then echoed some of de Cicco’s concerns regarding reimbursement for drug costs, noting the large disparities among the countries within Europe in the number of years a patient has to wait before any new drug become available. While the ECPC has been trying to standardise the amount of time patients wait to get access to drugs as well as requiring drugs be reimbursed within 100 days of treatment, more work still needs to be done to ensure equal access to all patients, regardless of the region or country.

The ECPC is also concerned with capacity building of organisations; they try to advance and disseminate activity to improve collaboration and networking. Their aim is to take care of all patients, including the long-time survivors and those in rehabilitation.

The sustainability of innovation in medical devices was the topic of Dr Claudio Viola’s talk on the Cliniporator technology, commonly known as electrochemotherapy. He noted that there are more effective health interventions than the National Health system (NHS) of Italy can afford (even avoiding wastes). The NHS has to choose which therapy to buy and which must be left. For this, a new approach must be proven to be selective, useful, and safe when compared to existing therapies. A budget impact analysis may also be performed to help decide if a new therapy or device will be worth its cost.

Electrochemotherapy, which was developed by a group of scientists in 2002, is such a technique that has demonstrated these requirements. There have been a number of papers published which look at the efficacy and cost comparisons of the treatment. There are now over 100 centres within Europe using electrochemotherapy, which can be used in skin metastases and advanced cases of melanoma.

Further comments on the importance of technological devices were then provided by Dr Eugenio Morsiani from RanD. He noted that the case of electroporation (referring to Dr Viola’s Cliniporator technology) was an example of Italian excellence in the world. He acknowledged that every patient is different and that one therapy cannot save all lives. Electroporation fills a gap. He said that all these available treatments are complementary, so why not apply a few techniques together, such as the electroporation and isolated limb perfusion. He agreed with Dr Viola in his hope that in the future the cost will decrease.

Following this last talk the speakers of the session were made available for a discussion. The cost of drugs and the time needed for drug approval were some of the first topics discussed. The need for randomised clinical trials for new technologies was also noted. Some also agreed that the restrictions placed on drug companies are sometimes severe and impartial.

The final talks of the first session featured two speeches from selected oral abstracts. Dr Simona Capporali looked at the invasion potential of cells with acquired resistance to the BRAF inhibitor. Specifically, she and her colleagues studied dabrafenib-resistant cells and the potential to block them by inhibiting the P13k/AKT/NF-kB pathway. She concluded from this study that the targeting of this pathway could be a good/efficient therapeutic strategy to inhibit not only the proliferation of cells with an acquired resistance to dabrafenib but also their invasive potential.

Dr Cristina Fortes then discussed the therapeutic effect of sentinel lymph node biopsy (SLNB) in melanoma. After a brief background on sentinel lymph nodes, Fortes detailed a retrospective cohort study on patients who had undergone an excision followed by a SLNB. They analysed the timing of the biopsy in relation to the overall survival of the patient. It was found that, for patients with a positive lymph node status, there was a benefit to undergoing a SLNB as soon as possible after excision.

### Session 2

The 2nd session opened with a talk by Maria Emilia Bonaccorso, an Italian journalist, who gave a talk entitled ‘Communication with patients: the goal of ethical journalism health’.

The point of view of insurance companies was then presented by Giuseppe Gionta. Dr Gionta started by providing a breakdown of Italian families with health policies and the type of policies taken out. He then focused on requirements for sickness policies. Oncology patients applying for insurance can fall into three categories: insurable without exclusions for a completely cured patient, insurable with exclusions, or non-insurable for those patients who have a serious illness.

### Session 3

The third session focused on some drug developments and started with a talk by BMS Oncology Medical Director, Dr CosimoPaga, on ipilimumab and programmed cell death (PD-1).

Dr Zaoit Szabo of Amgen GmbH, Switzerland, next discussed his work in oncolytic immunotherapy. In this treatment strategy, a tumour or lesion is injected with a virus, such as herpes simplex virus 1. It is believed that a viral infection can impact the immune system, which would then have an impact on cancer. There are many different virus and cancer types that have been investigated. Two of the most common cancers to be studied are melanoma and glioma and the leading viruses are the adenovirus and herpes simplex virus. Of seven clinical trials that used melanoma, in six of them the virus had a modified gene to express human granulocyte-macrophage colony-stimulating factor (GM-CSF), making them more selective for the tumour cells. Dr Szabo then discussed some early results from clinical trials. Crucially, in one trial they monitored lesions which were not injected with a virus. It was observed that the non-injected lesions showed a response through an immune-response.

Dr Loredana Orsini gave a talk on PD-1 and new drug projectsfrom Merck Sharp and Dohme which was followed by Dr Constantino Jemos of the IEO, who presented a talk on the management of new melanoma drugs in Italian hospitals from the IEO pharmacy. He looked at the costs of some popular drugs and discussed the savings strategies such as payment by results and drug days and the role that the pharmacy plays.

Dr Federico Pantellini from Roche outlined some of the work his company is doing in melanoma treatment. He looked at how treatments have evolved to become more individualised. He then focused on clinical trials involving vemurafenib and an anti PD-L1 drug and finally he briefly spoke about antibody drug conjugates.

Dr Alessandra Aloe from Merck Serono presented a talk on clinical research plans in melanoma. She spoke about their work with the MEK inhibitor pimasertib. They found that the drug has only limited benefit outside of melanoma and that drug resistance appeared fairly early. For this reason, the majority of their studies now look at pimasertib in combination with other therapies.

Next, Dr Eugenio Morsiani also spoke about clinical research plans in melanoma. His talk mainly focused on isolated limb perfusion (ILP). He highlighted a number of ILP clinical trials that used melphalan. He also pointed out that this technique is now accepted by a number of national bodies.

Dr Ruggerio Ridolfi, of Immunoterapia e Terapia Cellulare Somatica Meldola, gave a talk entitled ‘Planning ipilimumab therapy in a single institution’. He identified the Immunotherapy Unit and the Pharmacy Unit of the Romagna Cancer Centre as the focus of his talk. He noted that they were able to electronically follow drugs from prescription to the preparation of the drugs. He explained their results from a ‘Drug-day’ with ipilimumab (Yervoy®) in order to reduce the amount of unusable drug. This drug day has been going on since 2013 and they have improved in efficiency; 3 mg was unused in 2013, but 0 mg was unused in 2014. He noted that the average savings using the drug day was a little over 3 euros per mg, amounting to a savings of 7500 euro.

Dr Vanna Chiarion Silenimade had some comments on this talk. She addressed the issue of the relationship between effectiveness and efficacy. She also discussed economic results of centralisation in the Veneto region. They were able to recover money from both vial sharing and ‘payment by results’.

Dr Michele Guida of the Istituto Tumori Giovanni Paolo II, Brindisi, compared mono and combination treatments in MM. He noted that if combination therapy is employed, BRAF-inhibitor resistance will confer the resistance to the combination of drugs. Also, with combination therapy, there is a new toxicity profile and new side effects to consider.

Finally, Marianna Cavazza’s talk for the evening session looked at European Reference Networks, or allowing patients throughout Europe to choose where to be treated. She started by looking at the current legislation on patient travel and noted that cross-border inpatient care can be refused if the same treatment is available in the home country. She also discussed the terms under which the Italian NHS agreed to reimburse patients who had travelled within Europe for treatment. Of particular note was the idea that the costs would be reimbursed only up to the level of cost had the same treatment been performed in Italy.

### Session 4

Day two of the MIB conference started with a special focus on the patient viewpoint.

Dr Claudio Luchiari started the morning session with an impassioned talk on patient empowerment. He outlined the importance of the patient needing to be an active participant in choosing their treatment and the steps needed to ensure that patients are fully informed and capable to making their own decisions. The three key phrases Dr Luchiari stressed to ensure patient empowerment were active participation, partnership, between the doctor and patient, and self-management. Dr Luchiari noted that all people must take control of their health decisions as ‘citizens’, not only if and when they are patients.

Patient empowerment is more than just informed consent; the relationship between the health provider and patient changes. For this to happen, a patient must be fully informed and capable of making decisions on their treatment options. The role of the health provider is to make sure that the patient is in the right state of mind to understand and process information from the health provider. The patient must be able to integrate any prior information with what they learn later in order to make a decision. They need time to process information from both a cognitive and emotional perspective. Three points where patients need support from the treating team is in communication, access to research findings, and emotional support.

Hein Jambroers further stressed the importance of the patient perspective with his talk on his own personal experience as a cancer patient. He was diagnosed with melanoma in 2009 and after surgery was told he was cured. One year later, his melanoma had returned and he was told that he was untreatable. Mr. Jambroers discussed the highs and lows of being cured only to be diagnosed a year later and be told that he was incurable. He outlined the advice he received from Prof. Patricia Garcia-Prieto from Brussels and how that led him down the path to find treatment that ultimately led to his cure. Finally, as a cancer survivor, Mr Jambroers noted the changes in his professional and personal relationships and highlighted the importance of continued support for patients including when they are considered cured of their cancer.

Dr Paola Anabordi continued to focus on patients with a talk on the quality of life for patients. In particular, Dr Anabordi focused on the quality of life for patients in the chronic phase of the disease, or the extended survival phase. It has been shown that this phase is not well supported from a psychological perspective and studies have shown that patients still want information and support. They are implementing a phase I study based on interviews with patients in order to help make doctors understand how patients feel.

Prof. Claus Garbe of the University of Tuebingen in Germany switched the topic of the conference back to strategy when he outlined cancer centre certification procedures in Germany. A centre that has treated a pre-determined minimum number of patients for a type of cancer can apply to become certified for that particular organ. They first fill in a questionnaire and then undergo an initial inspection followed by annual rechecks. There are currently 41 certified skin tumour centres around Germany. Prof. Garbe then spoke about a white paper developed by the German Dermatologic Society and oncologic group. It was written over three years and contains 124 recommendations and statements.

Questions were raised about the uniformity of quality of care over the 41 certified centres, as well as if there minimum number of patients was high enough to ensure that the centre had the necessary degree of experience in that particular type of cancer.

Following on from Prof. Garbe’s talk, Dr Silvia Basso looked at cancer centre certification in Italy. She spoke about the different types of certification and accreditations available to the Italian centres.

Hein Jamroes then returned to provide some insight into how a patient chooses their hospital or doctor. For this, he first recalled his own attitude as a patient. He started fully trusting his doctors and performing only simple internet searches and as his disease progressed, started questioning his doctors more, recognising a good personality match, performing more specific searches, and then looking at patient groups on the internet. He noted that in the five years since he was diagnosed the online presence in patient organisations has increased dramatically.

Jamroes then provided a general overview of a patients’ attitude. A Stage I-II patient usually trusts their doctor but a Stage IV patient has a higher level of knowledge and expectations; they want a better quality of life and they expect it. Usually this patient would have done a fair amount of their own research and will actually need help managing all of the information that they have gathered. Emotion always plays a significant part in the patient’s actions and it is often difficult for them to separate their emotions from logical thought. It would be difficult for a patient to hear that they cannot have treatment because of it being too expensive.

A healthcare professional should be open to cutting edge research and must be open to a multidisciplinary approach and should be ready for questions from patients about what they read online. He finished by cautioning the healthcare professionals: you will be challenged by your patients.

### Session 5

Session 5 was discussion-based. Each speaker presented three slides to introduce a topic and encourage a discussion.

Prof. Nicola Mozzillo, of the Isituto Nazionaledei Tumori Pascale Napoli started the session with a brief presentation on when we can consider a patient clinically cured. He asked if the patient was cured after surgery and also looked at the methods employed, for example scans or bloodwork, to ensure a patient is disease free months or years later.

Dr Sara Vigna then looked at her life experience as a cured melanoma patient. Elisabetta Iannelli of the Anociazione Italianoper I Malatidi Cancro took the point of view of legislation and insurance companies and how she viewed it for cured patients. She asked if cured oncological patients can avoid being discriminated against by insurance companies. Dr Paolo Ascierto of the Istituto Nazionaledei Tumori Pascale Napoli spoke about his observation regarding management and financial resources in clinical trials. Finally, Carmen Verrengia spoke briefly about risk management and the quality of therapeutic outcomes.

### Session 6

The sixth session followed the same format as Session five, with speakers giving three slides to introduce a topic and initiate a discussion. Dr Giuseppe Palmieri gave a talk entitled ‘Genetic profiling in melanoma: a step to identify patients susceptibility to target therapy.’ Prof GeradoBotti of the Istituto Nazionaledei Tumori Pascale, Napoli presented some slides on ‘Target therapy molecular identification: role of quantitative evaluation.’ Dr Michele Maio of Policlinico Santa Maria alle Scotte, Siena, spoke about ‘Identifying patients susceptibility to immunotherapy: the clinical point of view.’ Finally, Prof. Paolo Ascierto returned to discuss ‘Mechanisms of resistance to target therapy.’

Following the second round table discussion, some time was devoted to updating the melanoma white paper, which had been published by the ECPC in 2007 [6]. Prof. De Lorenzo introduced the white paper by first thanking the organisers of the meeting for putting patients in such a central role. The white paper had been developed as a collaboration between melanoma patient Patricia Garcia-Prieto and ECPC. It was De Lorenzo’s hope to update it and produce different tools to inform the general cancer patient about organisations or to inform and support patients. The attendees then discussed various aspects of the paper and agreed to work on parts of it.

### Session 7

The final session of the meeting contained short presentations on technologies supporting health practice.

Prof. Carlo Ricardo Rossi (Istituto Oncologico Veneto, Padova) started the session with a talk on the some of the preliminary results from the Retemela Project. Retemela is an Italian acronym for melanoma network and its started in 2012 in Veneto, Italy. The project is still in its early stages. The aims of the project are to establish a network for the diagnosis first of pigmented lesions and then melanoma, to define a shared diagnostic and therapeutic pathway, to improve early diagnosis and shorten waiting lists, to improve the skills of the operators, to collect data for epidemiological purposes, and to rationalise the resources used.

Thus far they have gotten a shared clinical pathway both for lesions and melanoma. Waiting lists have shortened by involving more outpatient clinics in diagnosis. They have also started a melanoma registry and hope to expand the project to a larger region.

Dr Alessandro Di Stefani then gave a talk on mole mapping in early detection programmes of high risk patients. He started off identifying the challenges associated with diagnosis of high risk patients. He noted a few methods that can be used to monitor the high risk patient. Total body photography provides a high resolution image of the whole body and can identify new or changing lesions, reduce unnecessary biopsies, and encourages self-screening. Dermoscopy is a more diffuse technique that improves diagnostic accuracy up to 35% and also decreases the number of biopsies. A patient can be monitored with dermoscopy. Low risk patients, or those with only a few lesions, could have benign lesions left untreated while suspicious ones are simply removed. If there are multiple suspicious lesions, the patient may have a short-term clinical dermoscopic follow-up. Patients with multiple lesions could have an initial evaluation with a manual dermoscope from which the so-called signature pattern is determined. With this signature pattern, they can identify any ‘ugly ducklings’.

Di Stefani concluded by noting that we are beginning to move from a clinical pathological diagnosis to an area of clinical imaging diagnosis. The final decision needs to summarise all the complexities of the patient. Small mapping should take into consideration the individual clinical onset of the patient.

In a follow-up commentary, Dr Ignazio Stanganelli (IRST Meldola) pointed out that different devices may produce different images for the same mole. In the short and long-term follow-up the lesions could be described as different if imaged with two different devices. He also pointed out that some melanomas are slow-growing and may not be observed with follow-up periods of only eight months.

Dr Caterina Longo presented the next talk on confocal microscopy. Confocal Microscopy is a new non-invasive technique that can envision cells *in vivo* and is helpful in highlighting different patterns in many skin tumours. It is especially useful in a small subset of lesions (i.e. nodule-raised tumours). Sometimes it can catch lesions that are not suspicious from a dermascopic point of view and can also reduce the number of excisions of benign lesions. In short, confocal microscopy can be used in combination with a number of other techniques to reduce the number of unnecessary excisions and also to identify lesions. For population screening, dermoscopy is still the best choice. Mole mapping can then be performed on high-risk patients and confocal microscopy can be used between these two extremes to save on unnecessary excisions of benign lesions and also to catch very subtle melanoma.

Prof. Franco Di Filippo of Istituto Regina Elena, Roma then gave a cost-analysis of limb-perfusion. This technique may be used for unresectable metastatic lesions. Prof. Di Filippo pointed out that there are limited side effects to this technique as it is performed only in a limb. The treatment has been shown to be fairly effective: a total of 20 studies of unresectable patients—average complete response rate found was 67% with an overall response rate of 90%. It is possible to perform this more than once even if it is a rare event. Still, a second treatment had an interesting complete and overall response.

Prof. Di Filippo showed a cost analysis in terms of an incremental cost analysis ratio (ICAR). Through this, he admitted that limb perfusion was on of the more expensive forms of treatment and electrochemotherapy is probably one of the most effective treatments for the cost. However, limb perfusion has a need and should be applied when it is mandatory. Our first task should be to cure the patient, the rest comes later.

Dr Lorenzo Borgognoni (Azienda Sanitaria Firenze—Istituto Toscano Tumori), gave a short talk on the network model of cancer institute in the Tuscan region. In this model, each specialist involved in melanoma care is noted in the system and this helps to avoid competition. Having a network, rather than a single centre, where all the specialists are gathered, allows for widespread access in the territory with multidisciplinary teams. The aims of the Institute include homogeneity, appropriateness and continuity of care with widespread quality.

He also highlighted some of the achievements of the Institute. With the help of over 100 specialists from all over Tuscany they prepared melanoma guidelines in 2007, which were recently updated in 2012. They have a group which meets monthly to discuss difficult cases and they have developed a melanoma cancer registry covering all 12 of the Tuscan health units.

Dr Borgognoni hopes that the work they have done in this region, could contribute to future planning and the improvement of melanoma care.

The meeting concluded with talks from Dr De Lorenzo and Dr Testori, and it will be adjourned till 9–10 November 2015.
